# Does Life Satisfaction Mediate the Association between Socioeconomic Status and Excessive Internet Use?

**DOI:** 10.3390/ijerph16203914

**Published:** 2019-10-15

**Authors:** Laura Bitto Urbanova, Jana Holubcikova, Andrea Madarasova Geckova, Sijmen A. Reijneveld, Jitse P. van Dijk

**Affiliations:** 1 Department of Health Psychology, Faculty of Medicine, PJ Safarik University, Trieda SNP 1, 040 11 Kosice, Slovakia; jana.holubcikova@upjs.sk (J.H.); andrea.geckova@upjs.sk (A.M.G.); 2 Graduate School Kosice Institute for Society and Health, PJ Safarik University, Trieda SNP 1, 040 11 Kosice, Slovakia; s.a.reijneveld@umcg.nl (S.A.R.); j.p.van.dijk@umcg.nl (J.P.v.D.); 3 Olomouc University Social Health Institute, Palacky University in Olomouc, Univerzitni 22, 771 11 Olomouc, Czech Republic; 4 Department of Community and Occupational Medicine, University Medical Center Groningen, University of Groningen, 9713 AV Groningen, The Netherlands

**Keywords:** socioeconomic status, life satisfaction, excessive Internet use, adolescents

## Abstract

Excessive Internet use is becoming a rapidly increasing problem in today’s society. Our aim was to assess the association between socioeconomic status (SES) of the family and excessive Internet use (EIU), and whether life satisfaction mediates this association. We analyzed data from a representative sample of 2844 Slovak adolescents (mean age 14.34, 50.5% boys) from the 2014 Health Behavior in School aged Children (HBSC) study, based on self-report questionnaires. We assessed the association of SES, measured by several indicators, such as perceived family wealth, parental education, and (un)employment, and adolescent EIU using linear regression, adding life satisfaction as a mediator. Adolescents whose father was unemployed and whose perceived family wealth was low tended to score higher on EIU. Neither gender nor age affected this relationship. Life satisfaction mediated a part of the association between SES and EIU in the case of low perceived family wealth and father’s (un)employment. Adolescents with a low SES are more likely to become excessive Internet users, and life satisfaction mediates this association. Prevention of EIU among adolescents should be targeted at those with low SES, with life satisfaction being the topic to address.

## 1. Introduction

The Internet has many positive aspects supporting the life of adolescents, but it may become detrimental if it is used excessively. According to previous research, excessive screen time exposure can lead to an increase in an adolescent’s difficulties in falling asleep [1]. Furthermore, daily use of electronic media communication has been found to be associated with frequent online and also offline adolescent interactions with peers, which can consequently increase the probability of substance use [2]. Several attempts have been made to develop models and definitions of excessive Internet use and Internet addiction. This has led to many different terms used by different researchers to define excessive Internet use, such as pathological Internet use, problematic Internet use, or Internet addiction [3,4]. Davis [4] proposed a cognitive behavioural model, which explains pathological Internet use as the result of interaction between psychopathology and pathological Internet use, motivated by maladaptive cognitions users have about themselves. Furthermore, the model distinguishes two types of pathological Internet use: a specific one that includes people dependent on one online activity, and a general one, that reflects spending time online without specific objective, often motivated by the user’s need for social contact [4]. According to Muller et al. [5] and Montag et al. [6], there is a strong overlap between unspecified internet use disorder (IUD) and Internet communication disorder. However, in our study we focused only on unspecified excessive Internet use.

The other term used in this field is the IUD, which was developed in response to the inclusion of the Internet gaming disorder in Diagnostic and Statistical Manual of Mental disorders, 5th edition (DSM-5) [7,8]. According to An Interaction of Person–Affect-Cognition-Execution (I-PACE) model, IUD can be understood as a result of interaction between predisposing factors, such as psychopathology, vulnerable personality traits (P), affective (A) and cognitive (C) responses to the user’s experience with online application, executive and inhibitory control, and decision-making behavior that results in the addictive use of a specific Internet application [9]. To identify people reporting IUD, several criteria have been proposed, such as withdrawal, loss of interest in previous hobbies, loss of control according to games, tolerance, preoccupations, playing games to handle negative emotional states, lies about playing games, understanding the negative consequences of playing, and problematic relationships with relatives [8]. According to DSM-5 [8], users need to report five or more criteria from those mentioned above to be considered an Internet gaming addicts. In the current study, we use the definition of the EU Kids Online study [10], which assumes excessive Internet use to consist of components such as salience, tolerance, withdrawal symptoms, interpersonal and intrapersonal conflicts, and relapse. According to these components of excessive Internet use (EIU), there is some overlap between IUD criteria and EIU. Moreover, EIU also reflects the negative consequences and user’s feelings caused by their Internet use [3]. Using this definition, the EU Kids Online study [11] showed that 30% of the adolescents aged 11–16 years met the criteria for one or more components of EIU.

Adolescents from low socioeconomic status (SES) families are likely to have a rather high risk for EIU. Low SES of the family has been found to be associated with many difficulties in the lives of adolescents and with lower well-being [12]. In an attempt to deal with this situation, adolescents might start to use the Internet excessively. Research has shown EIU to be more likely among adolescents whose father’s education was lower and parental academic performance was poor [13]. Based on the previous findings of EU Kids Online, adolescents from families with lower SES are more likely to get upset if they are exposed to online risks [14]. Evidence is scarce on the association between SES and EIU using several indicators of SES. The few studies conducted on this topic have addressed only one or a limited number of SES indicators (e.g., only family income) and do not regard EIU as a possible precursor of addiction. Research is therefore needed on a wider range of SES indicators and on earlier stages of Internet addiction.

Regarding the potential mechanisms playing a role in the association between low SES and EIU, life satisfaction (LS) has been suggested as being part of that mechanism [15,16,17]. Low SES adolescents have rather poor LS, and this poor LS can result in a decrease in life engagement, and consequently focusing on the Internet. Previous research has shown negative associations between EIU and overall LS. In the case of specific types of LS, all facets of LS, such as health, job, income, dwelling, leisure, and family, have been found to be associated with EIU, with all associations significantly stronger for females [18]. Internet use can also serve as a possible compensation for negative affective states, such as stress, that have been found to be associated with a lower level of LS. According to previous findings, people who are vulnerable to reacting intensively to stress tend to report a higher level of EIU [19,20]. However, evidence is lacking on the associations between different indicators of SES and EIU, and whether LS mediates these associations. Therefore, the aim of our study was to explore the associations between different indicators of socioeconomic status of the family and EIU, and whether overall life satisfaction mediates these associations.

## 2. Materials and Methods

### 2.1. Sample and Procedure

We used data from the Health Behavior in School-aged Children (HBSC) study conducted in 2014 in Slovakia. In order to obtain a representative sample, we used a two-step sampling. First, 130 primary schools located in rural and urban areas from all regions of Slovakia were randomly selected from a list of all eligible schools in Slovakia and asked to participate in the study. The response rate (RR) of the schools was 86.1%. Second, from each school, one class of students per grade from the fifth to the ninth grades was asked to participate.

We collected data through a self-reported questionnaire. Participating students filled out the questionnaire in the class in the presence of a research assistant. The responses were anonymized. We obtained data from 9250 adolescents aged 11 to 15 years old (mean age = 13.48; 50.3% boys). In order to reduce the duration of administration, we used two types of questionnaires containing different sets of questions (equal in length but differing in the variables included). Those versions were randomly distributed within the sample. Both of them consisted of the mandatory questions that need to be included in HBSC questionnaires, and one set further contained questions on EIU. These questions on EIU were only administered to adolescents aged 13 to 15 years old. Thus, the final sample consisted of 2844 adolescents who responded to the optional package, including questions mapping EIU (mean age = 14.34; 50.5% boys).

The study was approved by the Ethics Committee of the Medical Faculty at P.J. Safarik University in Kosice (EC 09/2012). Parents were informed about the study via the school administration and could opt out if they disagreed with their child’s participation. Adolescents’ participation in the study was fully voluntary and confidential, with no explicit incentives provided for participation.

### 2.2. Measures

Data for the present study were collected using questionnaires from the standardized research protocols for the 2014 WHO-collaborative HBSC study. We obtained data on EIU, adolescent-reported SES of the family, and life satisfaction. We further assessed gender and age as potential confounding variables.

EIU was measured using five items with four-point Likert-type responses (never or almost never, not very often, fairly often, very often), which covered the five dimensions of Internet addiction [2]. Items were: salience (I have gone without eating and sleeping because of the Internet); tolerance (I have caught myself surfing when I am not really interested); withdrawal symptoms (I have felt bothered when I cannot be on the Internet); conflict (I have spent less time than I should with either family, friends, or doing schoolwork because of the time I spent on the Internet); and relapse (I have tried unsuccessfully to spend less time on the Internet). The EIU scale was created as the sum value of the above-mentioned five items, ranging from 5 to 20, with a higher score indicating a higher level of EIU. The psychometric properties of the EIU scale were shown to be satisfactory in the EU Kids Online study, which included adolescents in 25 European countries [21]. This study showed that only about 1% of European adolescents reported pathological use of the Internet [3]. Cronbach’s alpha in our sample was 0.69.

Socioeconomic status (SES) was measured by questions on perceived family wealth, mother’s highest education, father’s highest education, mother’s (un)employment, and father’s (un)employment. The perceived family wealth scale measured adolescents’ perception of their own family’s socioeconomic circumstances. Responses were: (1) not at all well off, (2) not so well off, (3) average, (4) quite well off, (5) very well off. Parental highest education was assessed at four levels: elementary school, apprenticeship, high school/gymnasium, university. Education was dichotomized into low (elementary school/apprenticeship, high school/gymnasium) and high (university) levels. We defined parental unemployment as (the lack of income due to) one or more parents not having paid work for various reasons, such as losing a job, staying at home because of health problems, studies, day care. This SES indicator was assessed by the question: Does your mother/father have a job? The responses were: yes, no.

Life satisfaction (LS) was measured using a Cantril ladder. Adolescents were asked how they would rate their life on a scale from 0 (worst possible life) to 10 (best possible life).

We used gender and age as potential confounding variables.

### 2.3. Statistical Analysis

First, we described the background characteristics of our sample: EIU, indicators of SES, and self-reported LS of adolescents. Before exploring the associations, we checked the model fit by assessing residual plots, variance inflation factors, and tolerance statistics. Then, we assessed the associations of each SES indicator separately with EIU, crude (Model 1), and adjusted for gender and age (Model 2) using ordinary linear regression. Next, we assessed the degree to which life satisfaction mediated the association between each SES indicator and EIU (Model 3). To explore whether the indirect effect of the independent variables on the dependent variable via the mediator was significant, we used the Sobel test. All data were analyzed using IBM SPSS Statistics 20.0 for Windows, and the Sobel tests were calculated via an online calculator available at http://quantpsy.org/sobel/sobel.htm.

## 3. Results

### Description of the Sample

Our respondents described their situation at home as quite well off. The majority of them reported a low parental education level. Moreover, 15.0% of the adolescents’ mothers and 6.6% of their fathers were unemployed (Table 1).

EIU was negatively associated with LS and perceived family wealth and positively associated with father unemployment and age in the total sample. For boys, the association between EIU and LS was r = −0.16, *p* = 0.000, and between EIU and family wealth r = −0.09, *p* = 0.001. In the case of girls, the association between EIU and LS was r = −0.20, *p* = 0.000, between EIU and perceived family wealth r = 0.10, *p* = 0.000, and between EIU and father (un)employment r = 0.07, *p* = 0.01 (Table 2).

According to the model fit, the histogram of standardized residuals showed that the data contained approximately normally distributed errors. The tests of collinearity statistics confirmed that multicollinearity was not a concern in our study. Furthermore, the test of analysis of variance showed that in the case of perceived family wealth, father’s unemployment, and parental education, the regression model significantly predicted the EIU.

Regarding the associations between various indicators of SES and EIU, we found that adolescents who perceived their family wealth as lower (β = −0.10) and whose fathers were unemployed (β = 0.23) tended to report higher EIU (Model 1, Table 3). Adjustment for age and gender hardly changed the association between SES and EIU (Model 2, Table 3). Furthermore, adolescents who reported a lower level of life satisfaction (β = −0.18) were more likely to score higher in EIU.

**Figure 1:**
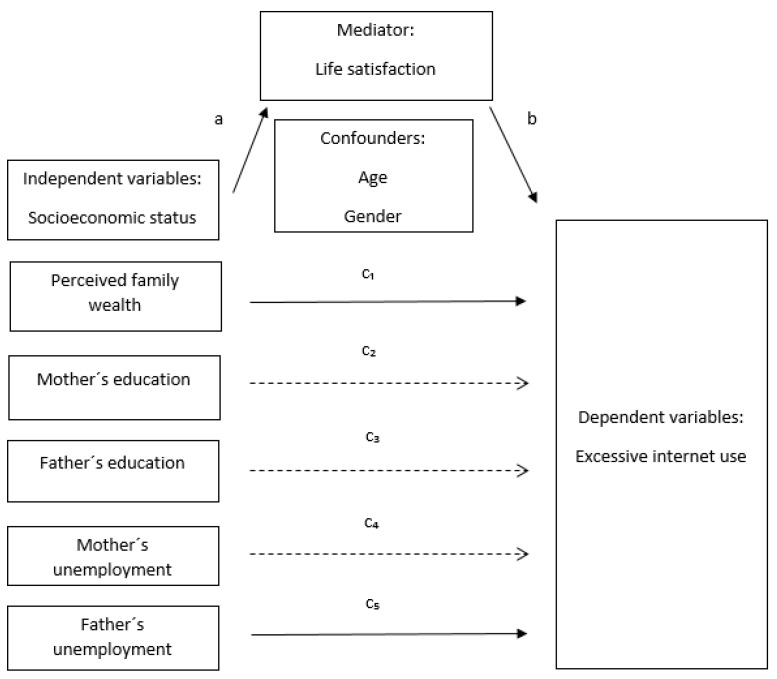
Conceptual model for the mediating effect of life satisfaction on the association between socioeconomic status and excessive Internet use. a = Association between indicators of socioeconomic status and the potential mediator, life satisfaction. b = Association between the mediator and excessive Internet use. C_1–5_ = Direct associations between indicators of socioeconomic status and excessive Internet use, adjusted for the mediator. 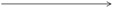 Confirmed mediation. 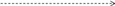 Not confirmed mediation.

The association between various indicators of SES (perceived family wealth and father’s (un)employment) and EIU as conceptualized in Figure 1 remained significant even after adjustment for life satisfaction (Model 3, Table 3). The outcomes of the Sobel tests confirmed the mediating role of life satisfaction on the association of SES with EIU regarding perceived family wealth and father’s unemployment (Table 4).

Below is a conceptual model that reflects the process of mediation confirmed in our results.

## 4. Discussion

We explored the associations of family SES with the EIU of Slovak adolescents and the potential mediating effect of LS on this relationship. We found that adolescents who perceived their family wealth as low and whose fathers were unemployed were more likely to score higher in EIU. Also, adolescents with a low level of LS tended to report a higher level of EIU. LS mediated the associations of several SES indicators (perceived family wealth and father’s unemployment) with EIU.

We found a lower level of SES to be associated with a higher level of EIU among adolescents, thus confirming previous findings [13,22,23]. However, in comparison to previous studies conducted on this topic, we used more SES measures than only family income. Our findings can be explained in several ways. First, the lower ability of low SES families to pay for organized leisure time activities for their children may contribute to a higher level of EIU. Children from low SES families tend to spend more time participating in unstructured leisure time activities (playing outside) [24]. As of 2018, more than 80% of Slovak households had access to the Internet [25]; thus, online activities represent one of the most affordable ways for adolescents to spend their free time. Second, the quality of parenting can affect the level of EIU, as it has been shown that having parental rules on spending time outside the house is associated with a decrease in adolescents’ screen time [26]. In Slovakia, half of parents of adolescents rarely or never apply rules on the computer use of their children, which can trigger EIU [27]. A lack of parental rules on adolescent Internet use can be explained by the lower Internet skills of low-educated parents [28]. Thus, EIU is more likely in low-SES adolescents, and the family environment, mainly parents’ education and unemployment, may play a significant role in their capacity to organize the time their kids spend using the Internet.

We also found that adolescents whose fathers were unemployed tended to report a higher level of EIU. Unemployment of parents is a stressful life event that can be considered one of the main reasons for a family crisis. According to previous findings, a father’s long-term unemployment can be associated with a lower level of adolescent well-being [29]. The father is often seen by his child as the head of family, as a person who is looking for the ways to meet family needs. Losing his job or long-term unemployment can cause negative emotional states in fathers and their children as well. Adolescents might adopt Internet use as an appropriate coping mechanism to handle this situation, which can lead to EIU.

We found that the association between low SES and EIU was partially mediated by LS. Previous research on LS as a mediator is fully lacking, as all previous research on the association between SES and EIU focused on the opposite pathway, i.e., that EIU can lead to lower LS [17]. Our finding could be explained in two ways. First, low SES adolescents may look for different ways to increase their LS and may use the Internet for this purpose. Internet use can also serve as a coping mechanism for mental health problems, such as depression, anxiety, or stress [30,31,32], which have been found to be associated with a lower level of LS [33,34]. If people prefer the use of the Internet in the case of negative emotional states instead of traditional coping strategies, this can lead to EIU [19,20,30]. A second explanation of our finding on the mediating effect of LS on the associations between SES and EIU is that online communication may represent a suitable environment for individuals to hide their low SES and to present themselves in a more desirable way. Adolescents reporting low LS may therefore prefer online communication, which subsequently leads to EIU [35]. In other words, when adolescents are trying to deal with the low SES of their family and poor LS, they can consider Internet use to be an appropriate way to handle their situation, as the online space can be seen as a place where they have the opportunity to control their self-presentation [36].

This study has several strengths, the major one being the large and nationally representative study sample of adolescents from 13 to 15 years old. Moreover, we used validated measures that have been used in a variety of reports before [14,37,38], and the setting of our study was confidential, with children filling out the anonymized questionnaires in the presence of a research assistant only. Some limitations should be mentioned as well. The first one is the cross-sectional design of the study, which does not allow final conclusions on the causality of associations between the variables used. Moreover, we used self-report questionnaires that can be influenced by social desirability. Adolescents can find it difficult to admit openly their level of Internet use and talk honestly about the SES of their family. However, the method of data collection is likely to have reduced this effect. Furthermore, in our study we focused only on cognitive facets of overall life satisfaction. Additional research on different domains of life satisfaction in the context of low SES might provide useful information for detecting risk factors for the development of EIU.

In our study we decided to measure EIU by using the EIU scale; however, our results imply a need for research focused on the correlation between EIU scale and other measures, such as Young’s Internet Addiction Test (IAT). Accordingly, our findings on the association between SES and EIU imply a need for preventive strategies that should focus on raising adolescents’ awareness about the benefits and risks of Internet use. This strategy should particularly target adolescents with a low SES, as they seem to be a vulnerable group. This study only focused on EIU in general, but further research on different phenomena associated with EIU (fear of missing out and problematic mobile phone use) and deeper analysis of the specific activities adolescents do online might provide useful information for designing effective strategies or interventions. Moreover, research is needed on interventions that can reduce the time spent on the Internet, with a reinforcing of real life activities among adolescents. A longitudinal study is needed to explore the causal pathways between SES and EIU.

## 5. Conclusions

This study demonstrated an association between lower SES of the family and the risk of EIU in adolescents, mediated by self-reported LS. Adolescents with lower SES in terms of lower perceived family wealth and an unemployed father reported lower LS and tried to find the way to deal with this unfavorable situation. Our study shows great potential to contribute to the development of preventive actions aimed at decreasing EIU. To support this, additional evidence should be collected, preferably with a longitudinal design that enables the clarification of causal pathways.

## Figures and Tables

**Table 1:** Background characteristics of the sample (2844 Slovak adolescents aged 13–15 years old, HBSC study 2014).

Variables	
Perceived family wealth (mean, SD) (range 1 (not at all very well off)–5 (very well off))	3.83 (0.83)
Mother’s education N (%)	
Low education	1663 (68.8)
High education	753 (31.2)
Father’s education N (%)	
Low education	1734 (75.2)
High education	572 (24.8)
Mother employment status N (%)	
Unemployed	409 (15.0)
Employed	2320 (85.0)
Father employment status N (%)	
Unemployed	173 (6.6)
Employed	2460 (93.4)
Life satisfaction (mean, SD) (range 0 (worst)–10 (best))	7.22 (1.93)
Excessive Internet use (mean, SD) (range 5 (low)–20 (high))	8.02 (3.13)
Gender N (%)	
Boys	1437 (50.5)
Girls	1407 (49.5)
Age (mean, SD) (range 13.00–15.92)	14.34 (0.80)

Missing values N (%): perceived family wealth 62 (2.2%), mother’s education 428 (15.0%), father’s education 538 (18.9%), mother unemployed 115 (4.0%), father unemployed 211 (7.4%), life satisfaction 52 (1.8%), excessive Internet use 280 (9.8%).

**Table 2:** Correlations between excessive Internet use (EIU), socioeconomic status (SES) indicators, demographic variables, and life satisfaction.

	Age	Life Satisfaction	Perceived Family Wealth	Mother Education	Father Education	Mother (Un)Employment	Father (Un)Employment
Total sample	0.04 *	−0.18 **	−0.09 **	−0.04	−0.04	0.01	0.06 **
Boys	0.03	−0.16 **	−0.09 **	−0.06 *	−0.02	0.01	0.04
Girls	−0.06 *	−0.20 **	0.10 **	−0.01	−0.06	0.01	0.07 *

* *p* < 0.05, ** *p* < 0.01.

**Table 3:** Associations between socioeconomic situation, life satisfaction, and potential confounders with degree of excessive Internet use, linear regression model adjusted for gender and age (2844 Slovak adolescents aged 13–15 years old, HBSC study 2014).

								Change of Model 2–3	
	Model 1		Model 2		Model 3		R^2^	R^2^ Change Model 2–3	β Change Model 2–3
	β (95% CI)	*p*	β (95% CI)	*p*	β (95% CI)	*p*			
Family wealth (1–5)	−0.10 (−0.14; −0.06)	***	−0.10 (−0.14; −0.06)	***	−0.05 (−0.09; −0.007)	*	0.011	0.024	0.05
Mother’s education	−0.08 (−0.17; 0.01)	ns	−0.07 (−0.16; 0.02)	ns	−0.05 (−0.14; 0.04)	ns	0.002	0.031	0.02
Father’s education	−0.09 (−0.19; 0.003)	ns	−0.09 (−0.19; 0.01)	ns	−0.06 (−0.15; −0.04)	ns	0.003	0.038	0.03
Mother (un)employment	0.03 (−0.08; 0.14)	ns	0.03 (−0.08; 0.14)	ns	−0.001 (−0.11; 0.11)	ns	0.002	0.033	0.03
Father (un)employment	0.23 (0.07; 0.39)	**	0.23 (0.07; 0.39)	**	0.20 (0.04; 0.36)	*	0.005	0.036	0.03
Life satisfaction (0–10)	−0.18 (−0.22; −0.14)	***	−0.18 (−0.22; −0.14)	***					

* *p* < 0.05, ** *p* < 0.01, *** *p* < 0.001. Model 1 crude model—effect of each SES indicator. Model 2 adjusted model—effect of each SES indicator after adjustment for gender, age. Model 3 adjusted model—effect of each SES indicator controlled for life satisfaction. Ns—Non significant. β change and R^2^ change between Model 2 and Model 3.

**Table 4:** The degree to which life satisfaction mediated the association between socioeconomic situation and excessive Internet use; results of the Sobel tests (2844 Slovak adolescents aged 13–15 years old, HBSC study 2014).

Path	Z	*p*-Value
family wealth →life satisfaction →EIU	−7.84	***
father (un) employment →life satisfaction →EIU	2.52	**

** *p* < 0.01; *** *p* < 0.001. EIU—excessive Internet use.
